# Giant Hiatal Hernia Mimicking Acute Coronary Syndrome: A Case Report

**DOI:** 10.7759/cureus.105653

**Published:** 2026-03-22

**Authors:** Mazen Mohandes, Mohamed Ghaith Al Abdin, Yousef M Alawi, Nazmus Saquib, Muhammed S Shahid

**Affiliations:** 1 College of Medicine, Sulaiman Al Rajhi University, Al Bukairiyah, SAU; 2 Department of Internal Medicine, Dr. Sulaiman Al Habib Medical Group, Al Qassim, SAU

**Keywords:** acute coronary syndrome mimics, cardiac biomarkers, chest pain, hiatal hernia, mediastinal compression

## Abstract

Hiatal hernia is a condition that involves the herniation of abdominal contents, most commonly the stomach, through the esophageal hiatus into the thoracic cavity. While often asymptomatic, large hiatal hernias can cause compression of mediastinal structures, producing symptoms that mimic acute coronary syndrome (ACS) such as chest pain, dyspnea, and elevated cardiac biomarkers. A 68-year-old woman with hypertension presented with five days of right-sided chest pain radiating to the back with nausea. Laboratory evaluation revealed elevated high-sensitivity troponin I (267.2 ng/L), pro-B-type natriuretic peptide (713 pg/mL), and D-dimer (1606 ng/mL fibrinogen-equivalent units (FEU)). Initial electrocardiogram showed non-specific findings. Chest X-ray demonstrated a retrocardiac opacity with gas-filled bowel loops and mediastinal shift, suspicious for a large hiatal hernia. Pulmonary embolism was ruled out via computed tomography (CT) pulmonary angiography. CT scan of the chest and abdomen confirmed a large hiatal hernia containing most of the stomach and part of the left colon. The patient was managed conservatively with intravenous fluids, serial cardiac biomarker monitoring, and thromboembolic prophylaxis. Her symptoms and biomarkers gradually improved, and surgical intervention was deferred due to age and comorbidities. This case highlights the diagnostic challenge posed by giant hiatal hernias mimicking ACS. Early recognition through imaging can prevent unnecessary invasive procedures, reduce healthcare costs, and guide appropriate management

## Introduction

The thoracic diaphragm, which separates the thoracic cavity from the abdominal cavity, contains openings, or “hiatuses,” that allow the passage of structures such as the esophagus and some neurovascular structures. In a hiatal hernia, the stomach protrudes through the esophageal hiatus and enters the thoracic cavity, where it could compress key cardiopulmonary structures [1]. The prevalence of hiatal hernia increases with age; approximately 55% to 60% of individuals older than 50 years are reported to have a hiatal hernia, and adults older than 70 years are more likely to present with large hiatal hernias. While hiatal hernias are relatively common, they rarely cause symptoms [2]. Only about 9% of patients develop symptomatic hiatal hernia. Symptoms are nonspecific and include gastroesophageal reflux disease (GERD), dysphagia, regurgitation, and chronic cough due to aspiration of undigested food [1]. However, a subset of patients present with atypical symptoms such as chest pain, dyspnea, palpitations, or chronic cough.

When a hiatal hernia is large enough to compress thoracic structures such as the heart, patients may present with symptoms that closely mimic acute coronary syndrome (ACS), either through coronary compression, direct myocardial compression, or supply-demand mismatch. Patients with ACS typically present with pressure-like retrosternal chest pain that may radiate to the jaw and or shoulder and is usually not related to respiration, palpation, or body movement. Moreover, troponin elevation and ischemic ECG changes increase concern for myocardial injury [3]. However, patients who are elderly, female, and patients with diabetes present with atypical symptoms (angina equivalents) such as dyspnea, diaphoresis, nausea and vomiting, and epigastric pain [4]. In these populations, ACS symptoms overlap with those of hiatal hernia, which may lead to diagnostic delay and uncertainty. This overlap may increase costs and patient anxiety because advanced and invasive diagnostic procedures, such as coronary angiography, are often considered.

In this report, we present a case of a large hiatal hernia presented to Dr. Sulaiman Al Habib Hospital, Al-Qassim, Saudi Arabia, with chest pain and elevated cardiac enzymes due to a hiatal hernia compressing the myocardial vessels. To our knowledge, there are fewer than 50 such cases reported in the literature, predominantly as isolated case reports. Furthermore, there are no cases of hiatal hernia mimicking ACS reported in Saudi Arabia. The goal is to analyze cardiac enzyme levels and form a differential diagnosis based on the clinical context as well as patient risk factors.

## Case presentation

A 68-year-old female with a history of hypertension and chronic exertional dyspnea presented to the cardiology clinic with right-sided atypical chest pain that radiated to the back for the past five days. She also complained of nausea but reported no acute shortness of breath at presentation and no change from her baseline chronic exertional dyspnea. She denied significant gastrointestinal complaints such as vomiting, diarrhea, or urinary tract symptoms such as dysuria, and denied fever. Her hypertension is managed with atenolol 50 mg once daily and captopril 25 mg twice daily. Additionally, she had a history of diabetic retinopathy and chronic exertional dyspnea of unclear etiology. There was no history of diabetes, myocardial infarction (MI), stroke, or other major comorbidities. On presentation, she was hemodynamically stable; vital signs were within normal limits except for tachycardia. Physical examination, including cardiovascular and respiratory assessment, was noncontributory.

**Figure 1:**
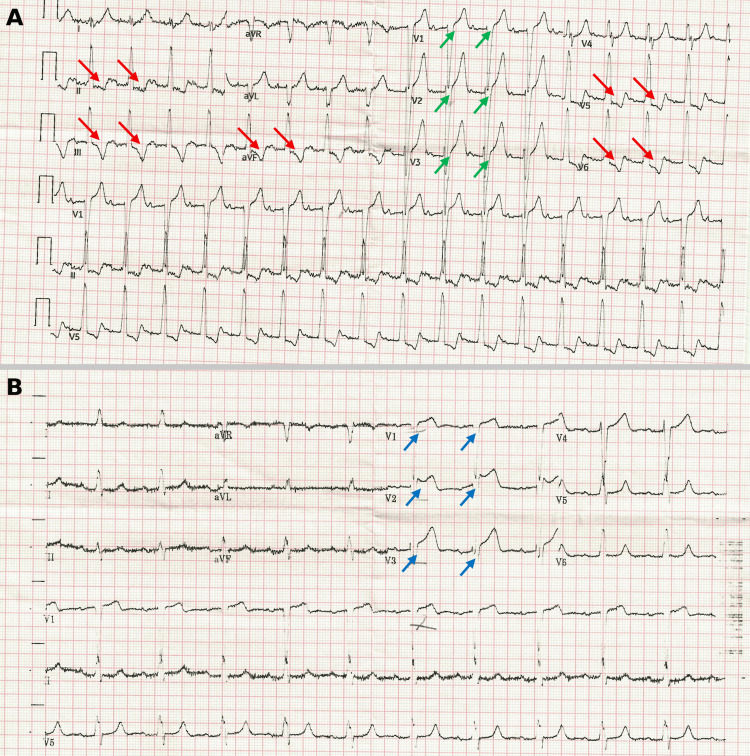
Admission and Serial ECGs (A) Admission ECG: Sinus tachycardia (102 bpm), prolonged PR interval (218 ms), deep asymmetrical T-wave inversions in leads II, III, aVF, V5, and V6 (red arrows), and ST elevation in V1–V3 (green arrows), consistent with left bundle branch block pattern and inferolateral ischaemic changes. No Sgarbossa-positive pattern was documented. (B) Serial ECG: Rate normalisation to 65 bpm, persistent first-degree atrioventricular (AV) block and left bundle branch block (LBBB), with improvement of the previously noted ST-T wave changes in the corresponding leads (blue arrows), consistent with improvement following conservative management of the underlying mechanical cardiac compression. It is acknowledged that the underlying LBBB limits complete ST-segment assessment by standard criteria.

**Figure 2:**
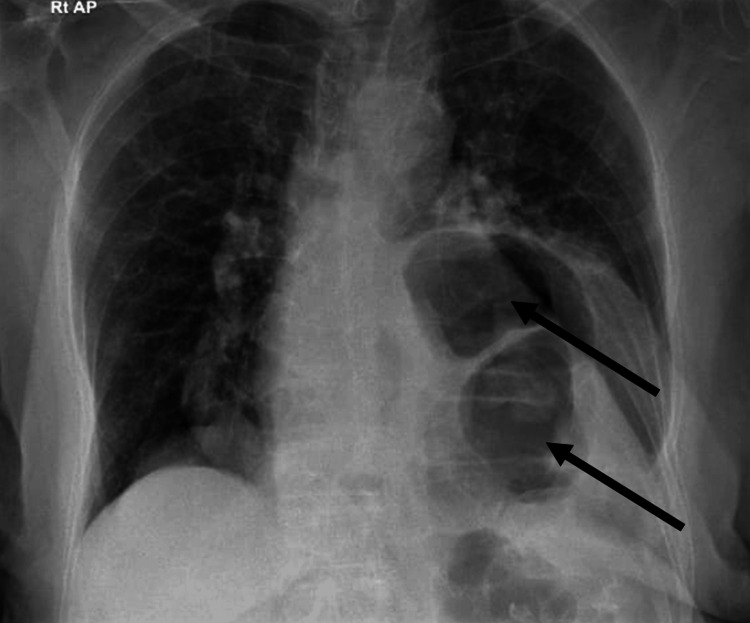
Posteroanterior Chest Radiograph Demonstrating Findings Consistent with Giant Hiatal Hernia. A large retrocardiac opacity is identified in the left hemithorax, containing gas-filled bowel loops representing herniated abdominal viscera (black arrows). Associated findings include leftward mediastinal shift and dorsal scoliosis convex to the right, reflecting the mass effect of the intrathoracic hernia on surrounding mediastinal structures. Reduced left lung volume secondary to compression by the herniated contents is also noted. These radiographic findings prompted further cross-sectional imaging to confirm the diagnosis.

**Figure 3:**
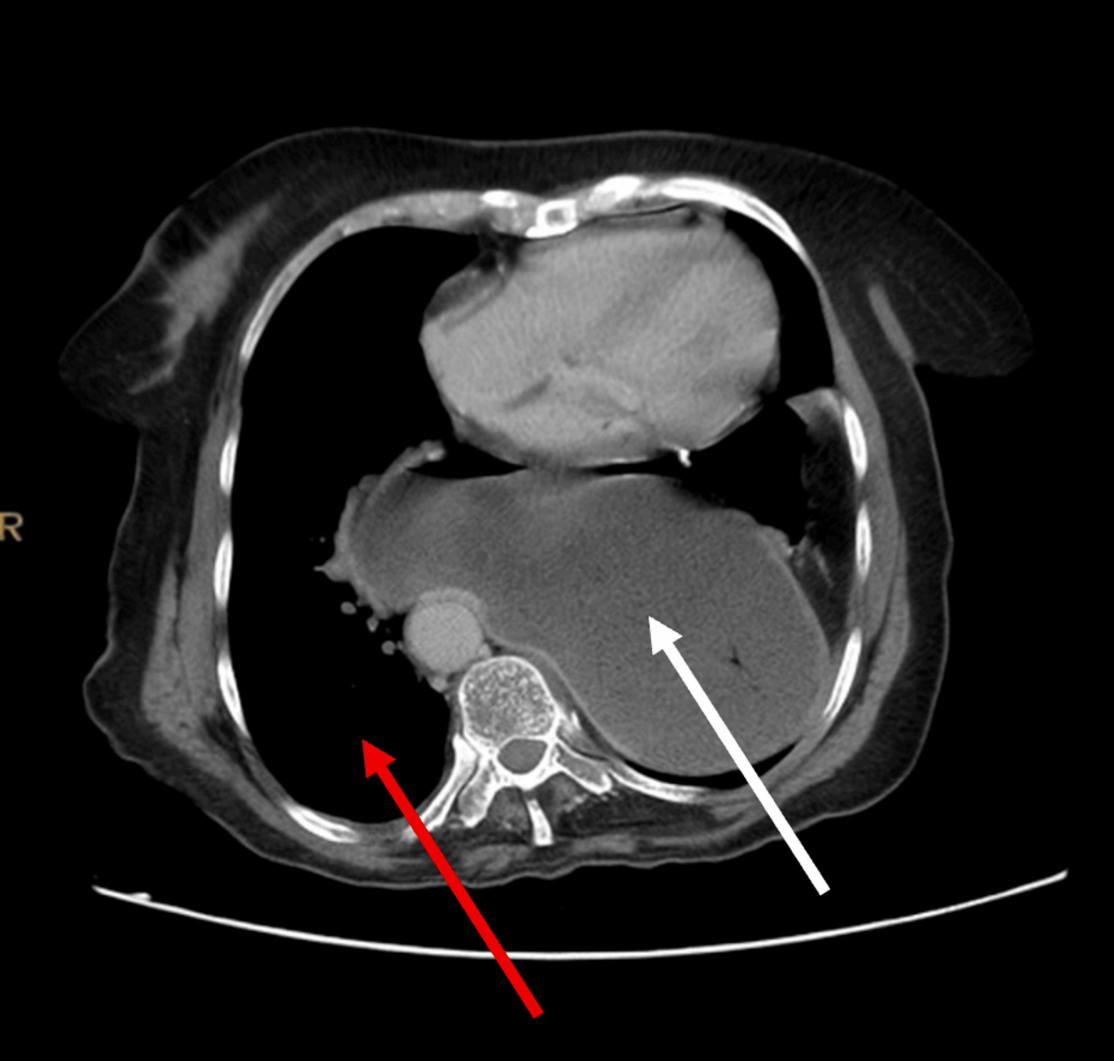
Axial CT Image of the Chest and Abdomen Confirming Giant Hiatal Hernia with Intrathoracic Herniation of Abdominal Viscera. Axial computed tomography (CT) image of the chest and abdomen with contrast demonstrating a giant hiatal hernia (neck diameter 5.2 cm) containing the majority of the stomach (white arrow) and a portion of the left colon (red arrow) herniated into the thoracic cavity posterior to the heart. The herniated viscera occupy a substantial portion of the left hemithorax, causing direct mechanical compression of the left ventricle and adjacent cardiac structures, reduced left lung volume, and leftward displacement of mediastinal contents.

Initial evaluation in the clinic revealed elevated serum biomarkers: high-sensitivity troponin I (hs-TnI) at 267.2 ng/L (normal range <15.6 ng/L), pro B-type natriuretic peptide (pro-BNP) at 713 pg/mL (normal range <125 pg/mL), and D-dimer at 1606 ng/mL fibrinogen equivalent units (FEU) (normal range <500). Her initial lab tests are presented in Table 1. Her ECG showed sinus tachycardia with a heart rate of 102 beats per minute, a prolonged PR interval (218 milliseconds), deep asymmetrical T-wave inversion in leads II, III, aVF, V5, and V6, and ST elevation in V1-V3 consistent with left ventricular hypertrophy (LVH) (Figure 1A). Given the conduction abnormality, the tracing was interpreted cautiously, and no Sgarbossa-positive pattern was documented. A posterior-anterior chest X-ray revealed a large retrocardiac opacity with gas-filled bowel loops in the left hemithorax, causing mediastinal shifting and dorsal scoliosis convex to the right side, findings concerning for a large hiatal hernia (Figure 2). A subsequent confirmatory CT scan of the chest and abdomen revealed a large hiatus hernia (neck diameter = 5.2 cm), causing decreased left lung volume, which contributed to the exertional dyspnea. The hiatal hernia contained most of the stomach and part of the left colon (Figure 3). Elevated D-dimer levels prompted a CT pulmonary angiography to rule out the possibility of a pulmonary embolus, which was negative for major pulmonary embolism. Because invasive coronary angiography or coronary CT angiography was not performed, obstructive coronary artery disease could not be excluded with absolute certainty. However, the decision to defer coronary angiography was based on the combination of clinical findings: the CT scan provided a compelling mechanical explanation; the serial troponin trend was non-escalating and inconsistent with acute Type 1 MI; the symptom pattern was atypical; and the procedural risk in this elderly patient was considered to outweigh the diagnostic yield in the context of a clear alternative diagnosis on non-invasive imaging. In the absence of thromboembolism, the elevated D-dimer was considered non-specific and may reflect age-related elevation and acute stress-related elevation associated with the intrathoracic herniation and cardiopulmonary strain.

**Table 1:** Initial Serum Cardiac Biomarker Results at Presentation Laboratory investigations obtained at initial evaluation demonstrating elevation of all three cardiac biomarkers above their respective reference ranges. High-sensitivity troponin I (hs-TnI) was elevated at 267.2 ng/L (reference <15.6 ng/L), representing an approximately 17-fold elevation above the upper limit of normal, consistent with myocardial injury. Pro-B-type natriuretic peptide (pro-BNP) was elevated at 713 pg/mL (reference <125 pg/mL), reflecting elevated ventricular filling pressures attributable to impaired diastolic function secondary to extrinsic cardiac compression. D-dimer was elevated at 1606 ng/mL FEU (reference <500 ng/mL FEU), prompting CT pulmonary angiography to exclude pulmonary embolism, which was negative; the elevation was subsequently attributed to non-specific acute cardiopulmonary stress and age-related factors. Abbreviations: FEU, fibrinogen equivalent units; ng/L, nanograms per litre; pg/mL, picograms per millilitre; ng/mL, nanograms per millilitre.

Initial Lab Test	Result	Reference Range
hs-TnI	267.2 ng/L	<15.6
D-dimer	1606 ng/mL FEU	<500
Pro-BNP	713 pg/mL	<125

Given these imaging findings and overall clinical picture, the patient's symptoms were determined to be primarily related to the large hiatal hernia compressing the left lung and heart, leading to respiratory symptoms, discomfort, and elevated cardiac markers, not due to an underlying cardiac disease.

The patient was managed with intravenous normal saline with dextrose, serial troponin level measurement, and continuous ECG monitoring to track for any changes in cardiac rhythm or ischemia. She was also advised on a low-fat cardiac diet. Over the next few days of management, her ECG findings improved (Figure 1B) and her cardiac enzyme levels decreased steadily, indicating no further myocardial injury (Table 2). Low-molecular-weight heparin (LMWH) was administered as standard inpatient venous thromboembolism prophylaxis during hospitalization and for reduced mobility. Blood pressure management was continued with her baseline antihypertensive regimen of atenolol and captopril.

**Table 2:** Serial Cardiac Troponin Measurements During Admission Demonstrating a Declining Trend The high-sensitivity troponin I (hs-TnI) demonstrated a non-escalating pattern followed by a steady decline over the course of admission, a kinetic profile inconsistent with the expected rise-fall pattern of acute Type 1 myocardial infarction and more consistent with reversible myocardial injury secondary to the mechanical compression of the left ventricle by the herniated viscera. Abbreviations: N, Normal Reference Range; ng/L, nanograms per Liter.

Day	Troponin I level (ng/L) (N <15.6 ng/L)
Day 1	267.2
Day 2	156.2
Day 3	73.4
Day 4	62.1
Day 5	53.0

During the course of her hospitalization, the patient's symptoms gradually improved with supportive care, and no further evidence of myocardial injury or thromboembolic events was noted. The surgical team was consulted and advised continued conservative management due to her high troponin levels which indicate high cardiovascular risk.

## Discussion

Although most hiatal hernias are asymptomatic, large hiatal hernias can present with typical symptoms of GERD, early satiety, nausea, bloating, and chest/abdominal pain. However, a subset of individuals present instead with extraintestinal symptoms, making diagnosis in these patients particularly difficult [5]. Such presentations have been described as gastro-cardiac syndrome (Roemheld syndrome) and may manifest as myocardial injury or Type 2 MI due to supply-demand mismatch and/or mechanical compression with resulting damage to the epicardium. The resulting supply-demand mismatch is consistent with a Type 2 MI mechanism as defined by the Fourth Universal Definition of Myocardial Infarction [6], whereby extrinsic compression rather than coronary thrombosis drives cardiomyocyte injury and troponin release. Supporting this interpretation, the serial hs-TnI in our patient demonstrated a non-escalating and subsequently declining trend, suggesting reversible mechanical injury rather than ongoing ischaemic necrosis.

A hiatal hernia can get large enough to allow the stomach to protrude through it and compress the myocardial vessels, mimicking a much more sinister diagnosis, a myocardial infarction. Even though the underlying mechanism is not fully understood, multiple mechanisms have been postulated [7]. When the stomach protrudes into the thoracic cavity, it lies posterior to the mediastinum. The stomach exerts pressure on the mediastinum and causes the heart to shift clockwise with resultant right axis deviation and corresponding ECG changes as well as elevated pro-BNP [8,9]. The herniated stomach could also compress the vagal nerve, which causes vagal nerve overstimulation [10]. ECG changes include sinus bradycardia, sinus pauses, and atrioventricular (AV) blocks [11]. Lastly, ECG changes such as widespread ST-segment elevation and depressed PR interval can be caused by pericardial irritation due to contact with the herniated abdominal content [9].

Marked elevation of high-sensitivity troponin indicates myocardial injury rather than isolated autonomic symptoms. In the setting of a large hiatal hernia and the absence of coronary artery luminal obstruction, plausible mechanisms include Type 2 MI from transient supply-demand mismatch, direct epicardial/pericardial irritation, or mechanical compression of the myocardium and coronary vasculature [12]. D-dimer is a sensitive but non-specific marker that can be elevated in the elderly and in many inflammatory or stress states. In this case, it appropriately triggered evaluation for pulmonary embolism. Once excluded, the elevation was interpreted as non-diagnostic and potentially related to acute illness and cardiopulmonary strain from the intrathoracic herniation [13].

Elevated troponin I levels can be due to cardiac and non-cardiac causes. Non-ST-segment elevation myocardial infarction (STEMI) cardiac causes of elevated troponin I level include aortic dissection, arrhythmias, heart failure, chest trauma, and inflammation such as viral myocarditis [14]. Non-cardiac causes of elevated troponin I level include chronic kidney failure, acute pulmonary embolism, strenuous exercise, acute pancreatitis, and critical illness in the Intensive Care Unit [15]. Alternative diagnoses were systematically excluded. Pulmonary embolism was ruled out by CT pulmonary angiography. Pericarditis was considered but excluded by the absence of pleuritic pain, positional features, and typical saddle-shaped ST elevation. Type 1 MI, the primary initial concern, could not be definitively excluded because coronary angiography was not performed; however, it was considered less likely given the atypical pain distribution, non-escalating serial troponin trend, lack of a Sgarbossa-positive ECG pattern, and identification of a convincing mechanical aetiology on cross-sectional imaging.

A handful of cases have been reported on large hiatal hernias compressing the mediastinal structures and causing myocardial infarction with non-obstructive coronary arteries (MINOCA) (Table 3). There is a variety of mechanisms by which MINOCA can occur. The initial workup of these cases included an ECG, chest X-ray (CXR), and subsequently a coronary angiogram to rule out a STEMI. It was after these investigations were negative that a noncardiac cause was suspected and a CT scan was ordered, revealing a large hiatal hernia behind these symptoms [7,16].

**Table 3:** Summary of Published Cases of Giant Hiatal Hernia Mimicking Acute Coronary Syndrome, Including the Current Case. In the majority of published cases, coronary angiography was performed and demonstrated non-obstructive coronary arteries, fulfilling criteria for MINOCA or Type 2 MI. In the present case, coronary angiography was deferred based on the compelling alternative mechanical diagnosis established on cross-sectional imaging, the non-escalating serial troponin trend, and a careful risk-benefit assessment; conservative management resulted in clinical and biochemical improvement. This table is provided as a differential diagnostic reference and does not imply that the current patient met formal diagnostic criteria for MINOCA, which requires angiographic confirmation. Abbreviations: ACS, acute coronary syndrome; MI, myocardial infarction; MINOCA, myocardial infarction with non-obstructive coronary arteries; CTPA, CT pulmonary angiography; hs-TnI, high-sensitivity troponin I; FEU, fibrinogen equivalent units; CT, computed tomography.

Report	Patient	Cardiac-mimic features / Key investigations	Management / Outcome
Current case	68 Female	Chest pain; elevated high-sensitivity troponin levels 267.2 ng/L; D-dimer 1606 ng/mL FEU; CTPA negative; CT chest/abdomen: giant hiatal hernia (stomach + left colon)	Conservative; biomarkers improved; surgery deferred
Narala K et al. [16]. Hiatal hernia mimicking ST elevation myocardial infarction.	72 Female	ST-elevation MI mimic; hiatal hernia identified on imaging after noninvasive and invasive cardiovascular workup	NR
Rubini Gimenez M et al. [7]. A case report of a giant hiatal hernia mimicking an ST-elevation myocardial infarction.	61 Female	ST-elevation MI mimic; after bicycle exercise testing, she developed acute chest pain and her ECG showed significant ST-segment elevations. High-sensitivity cardiac troponin was elevated, CT demonstrated giant hiatal hernia	Giant hernia was surgically corrected, and the patient’s exertional dyspnoea fully relieved during follow-up

In patients with exertional dyspnea and atypical chest pain, it is essential to consider extra-cardiac causes of ST changes on ECG and elevated troponin I early in the workup to avoid delays in diagnosis and treatment, as well as to reduce the costs of unnecessary workup.

## Conclusions

This case illustrates that giant hiatal hernias may mimic ACS through a constellation of chest pain, elevated cardiac biomarkers, and ECG abnormalities consistent with a Type 2 myocardial injury mechanism, whereby extrinsic cardiac compression produces supply-demand mismatch without underlying coronary occlusion. While the clinical and biochemical improvement observed with conservative management strongly supports a mechanical aetiology in this case, the absence of coronary angiography means obstructive coronary artery disease cannot be excluded with absolute certainty. Physicians should remain mindful of rare extracardiac causes of elevated biomarkers and ECG changes, particularly in elderly patients presenting with atypical chest pain and an inconclusive initial cardiac workup. Early cross-sectional imaging plays a pivotal role in identifying such aetiologies, potentially avoiding unnecessary invasive procedures and reducing associated procedural risk. A multidisciplinary approach involving cardiology, radiology, and surgery remains essential to optimise patient outcomes in these diagnostically challenging presentations.
